# Preparation, characterization, antimicrobial and cytotoxicity studies of copper/zinc- loaded montmorillonite

**DOI:** 10.1186/s40104-017-0156-6

**Published:** 2017-03-21

**Authors:** Lefei Jiao, Fanghui Lin, Shuting Cao, Chunchun Wang, Huan Wu, Miaoan Shu, Caihong Hu

**Affiliations:** 1 Animal Science College, Zhejiang University, Key Laboratory of Animal Feed and Nutrition of Zhejiang Province, No.866, Yuhangtang Road, Hangzhou, 310058 People’s Republic of China; 2 Key Laboratory of Animal Nutrition and Feed in East China, Ministry of Agriculture, No.866, Yuhangtang Road, Hangzhou, 310058 People’s Republic of China

**Keywords:** Antimicrobial reagent, Modified montmorillonites, Synergistic antimicrobial effect

## Abstract

**Background:**

A series of modified montmorillonites (Mt) including zinc-loaded Mt (Zn-Mt), copper-loaded Mt (Cu-Mt), copper/zinc-loaded Mt with different Cu/Zn ratio (Cu/Zn-Mt-1, Cu/Zn-Mt-2, Cu/Zn-Mt-3) were prepared by an ion-exchange reaction, and characterized using X-ray diffraction (XRD), fourier transformed infrared spectroscopy (FTIR) and transmission electron microscopy (TEM). The specific surface areas, antimicrobial activity and cytotoxicity of the modified Mt were investigated.

**Results:**

In the modified Mt, hydrated Cu ions and Zn ions were exchanged in the interlayer space of Mt and the particles were irregular shapes. The results showed that Cu/Zn-Mt enhanced antibacterial and antifungal activity compared with Zn-Mt and Cu-Mt possibly due to the synergistic effect between Cu and Zn. Among the Cu/Zn-Mt with different Cu/Zn raitos, Cu/Zn-Mt with a Cu/Zn ratio of 0.98 or 0.51 showed higher antimicrobial activity against gram-negative bacteria (*Escherichia coli*), gram-positive bacteria (*Staphylococcus aureus*)*,* fungi (*Candida albicans*)*.* Moreover, the antimicrobial activity of Cu/Zn-Mt was correlated with its specific surface area. Cytotoxicity studies on IPEC-J2 cell showed a slight cytotoxicity of Cu/Zn-Mt.

**Conclusions:**

The current data provide clear evidence that in terms of its antimicrobial activity and relatively low toxicity, the Cu/Zn-Mt holds great promise for applications in animal husbandry.

## Background

Recently, various inorganic antimicrobial materials have been developed and have attracted considerable interest in animal husbandry [1–3]. Among various antimicrobial metals,Copper (Cu) and Zinc (Zn) are normally used in animal feed in concentrations in excess of the nutritional requirements of the animals and for prevention of diarrhea disease, and also as an alternative to in-feed antibiotics for growth promotion [4, 5]. However, the strategy has been criticized because high level of Zn and Cu give rise to microbial drug resistance [6–8]. Enteral bacteria, both commensal and pathogenic, in farmed animals have been shown to develop resistance to trace elements (Cu and Zn) and concomitant cross-resistance to antimicrobial agents. Such bacteria may be transferred to other animals and human [7]. Moreover, large quantities of Cu/Zn were excreted, which would pose an environmental problem [4]. Therefore, it is essential to find an alternative to reduce Cu/Zn supplementation for sustaining animal production.

Special attention has been paid to metal ions loading inorganic carrier, which are superior in terms of safety, and long-term antibacterial effectiveness when compared with conventional metal [9–11]. As for inorganic carriers, the use of clay minerals as supports for synthesis of various inorganic antimicrobial materials has attracted considerable interest, owing to their nontoxic, environmentally friendly characteristic, and easy preparation [12–15]. It has been demonstrated that heavy metal (silver, Cu, Zn, and so on) exchanged clay minerals can serve as an antimicrobial reagent in vitro [8, 10, 16]. Until recently, Cu or Zn exchanged clay minerals has been added to the animal feed as an antibiotic alternative [4, 8, 15], with the additive amount of Cu and Zn being quite lower than that in conventional animal diet. Moreover, a few researches have reported that loading two metal ions onto montmorillonite (Mt) displayed obvious synergistic antimicrobial effect in vitro [17–19]. However, there are no data loading Zn^2+^ and Cu^2+^ onto Mt so far. It has been reported that Cu-Mt and Zn-Mt displayed different antibacterial and antifungal activity [10]. Mt with different metal loading capacity had different physical and chemical properties [20–22]. So whether the Cu/Zn- loaded Mt (Cu/Zn-Mt) with different Cu/Zn ratios will affect the antibacterial and antifungal effect in vitro or not need to been explored.

Moreover, since the ultimate location of Cu/Zn-Mt is the intestinal tract of animals, it is necessary to test its cytotoxicity using an in vitro model for safety consideration. Recently, a cell line from jejunum epithelium isolated from a neonatal unsuckled piglet, small intestinal porcine epithelial cell line (IPEC-J2) was characterized and used as an in vitro model system for study [23]. This cell lines exhibited strong similarities to primary intestinal epithelial cells, and can be used as an appropriate model through the advantage of direct comparison with the experimental animals [24]. Therefore, IPEC-J2 was selected for the cytotoxicity studies of Cu/Zn-Mt.

In the present work, a series of modified Mt including Zn-Mt, Cu-Mt, Cu/Zn-Mt with different Cu/Zn ratio (Cu/Zn-Mt-1, Cu/Zn-Mt-2, Cu/Zn-Mt-3) were synthesized. Their synergistic antibacterial and antifungal effect in vitro were compared*.* Considering the application of Cu/Zn-Mt in animal husbandry, cytotoxicity assay was measured, too.

## Methods

### Materials

Mt was obtained from the Inner Mongolia Autonomous Region, China. The content of the purified Mt was 99.0%. The cation exchange capacity (CEC) was 1.30 mmol/kg Mt. ZnSO_4_.7H_2_O and CuSO_4_.5H_2_O were purchased from Sinopharm Chemical Reagent Co., Ltd., China. Gram-negative *Escherichia coli* (*E. coli*, ATCC 25922), gram-positive *Staphylococcus aureus* (*S. aureus*, ATCC 29213) and *Candida albicans* (*C. albicans,* ATCC 10231, fungi) were purchased from China Center of Industrial Culture Collection.

### Preparation of modified Mt

The Mt (Cu-Mt, Zn-Mt, Cu/Zn-Mt-1, Cu/Zn-Mt-2, Cu/Zn-Mt-3) were prepared by an ion-exchange reaction. Ten grams of the Mt was mixed with 0.1 L of 0.2 mol/L NaCl solution. The dispersion was agitated for 5 h on a magnetic stirrer (700 rpm). The Na-Mt was then separated by centrifugation (15 min, 8000 × g) and washed with deionized water for three times. The washed Na-Mt was then added to 0.1 L of 0.19 mol/L ZnSO_4_ solutions, 0.2 mol/L CuSO_4_ solutions, 0.2 mol/L CuSO_4_ and ZnSO_4_ mixed solutions (the ratio of Cu and Zn is 1:1, 1:2, 1:4), respectively. The dispersion was agitated at 60 °C for 6 h on a magnetic stirrer to accelerate the cation exchange. After centrifugation, the sediment was washed with deionized water for three times, dried at 80 °C over night, and ground to a size less than 300 mesh. Zinc or/and copper concentration in the modified Mt were measured by atomic absorption spectroscopy (ICE 3300, Thermo Fisher Scientific, Waltham, USA). The Cu concentration of Cu-Mt was 5.70%. The Zn concentration of Zn-Mt was 5.62%. The Cu and Zn concentration of Cu/Zn-Mt-1 were 2.78%, 2.85%, respectively. The Cu and Zn concentration of Cu/Zn-Mt-2 were 1.89%, 3.72%, respectively. The Cu and Zn concentration of Cu/Zn-Mt-3 were 1.15%, 4.45%, respectively. .

### Characterization of the specimens

A PANalytical X'pert PRO powder diffractometer equipped with a Cu Kα radiation source was employed to determine the phase compositions and structures of the samples at 40 kV and 30 mA. Fourier transform infrared spectrometer (FTIR) was performed with a Nicolet Avatar 37- DTGS FT-IR spectrophotometer to study the structure of the materials by analyzing the vibrational frequencies of chemical bonds. Transmission electron microscopy (TEM) and energy-dispersive x-ray spectroscopy (EDX) (Tecnai G2 F20 S-TWIN; FEI Company, Hillsboro, OR, USA) were carried to characterize the microstructure of the samples.

### Antimicrobial activity of the specimens

For antimicrobial experiments, the minimum inhibitory concentrations (MIC) of the specimens were estimated by a two-fold diluting method. The typical microorganisms of *E. coli*, *S. aureus* and *C. albicans* were selected as indicators. Luria Bertani (LB) broth was used as a growing medium for *E. coli* and *S. aureus. C. albicans* was cultivated in liquid sabouraud medium. Bacterial strains were grown overnight and diluted with fresh medium to achieve an approximate density of 10^7^ CFU/mL. Specimens of each material (Mt, Cu-Mt, Zn-Mt and Cu/Zn-Mt) were put into tubes containing 5 mL LB broth or liquid sabouraud medium, and then two-fold diluted into different concentrations. Bacterial inoculums were added to tubes with a final concentration of 10^5^cells/mL. Each specimen was determined in triplicate. The bacteria–mineral mixtures were incubated at 37 °C for 24 h, with continuous shaking at 200 rpm. The MIC of the specimens was determined by the lowest concentration of the specimens that inhibited completely the bacteria or fungi visible growth when judging by eye [25].

### Particle size, specific surface areas and antimicrobial assays of Cu/Zn-Mt

Cu/Zn-Mt was ground to a size less than 100, 200, 300, 400 mesh, respectively. The N2 adsorption isotherms and specific Brunauer-Emmitt-Teller (BET) surface areas of Cu/Zn-Mt were measured at 77 K, using a Tristar 3000 specific surface area and porosimetry analyzer (Micromeritics Instrument Corp., USA). All the samples were degassed at 623 K for 1 h under vacuum before analysis.

For antimicrobial assays of Cu/Zn-Mt with different article size and specific surface areas, Cu/Zn-Mt was put into tubes containing nutrient broth to achieve a concentration of 400 mg/L, and then the bacterial inoculums (*E. coli* or *S. aureus* or *C. albicans*) were added with an approximate density of 10^5^cells/mL. The tubes without Cu/Zn-Mt served as the control group. After incubation at 37 °C for 24 h, the mixtures were subjected to successive 10-fold serial dilutions in the corresponding medium, mixed with a vortex shaker to ensure dispersion and quantitatively cultured in duplicate onto agar plates to determine the number of viable bacteria. The viable cell counts were expressed as fold changes, calculated relative to the control group.

### Cytotoxicity assay of Cu/Zn-Mt

Cytotoxicity of Cu/Zn-Mt was performed on IPEC-J2 cell by tetrazolium dye (MTT) based assay. Briefly, cells were seeded into 96-well culture plate in triplicate (1.2 × 10^5^ number of cells in 100 μL DMEM). Mono-layers of cells were treated with Cu/Zn-Mt of increasing concentration (0–0.5 mg/mL). At the end of the culture period, 20 μL of 5 mg/mL MTT stock solution was added in each well. After additional 4 h of incubation at 37 °C, the resultant intracellular formazan crystals were solubilized with acidic isopropanol and the absorbance of the solution was measured at 570 nm using an ELISA reader (Emax, Molecular device, CA, USA).

## Results and Discussions

### Structure and morphology analysis of the specimens

**Fig. 1:**
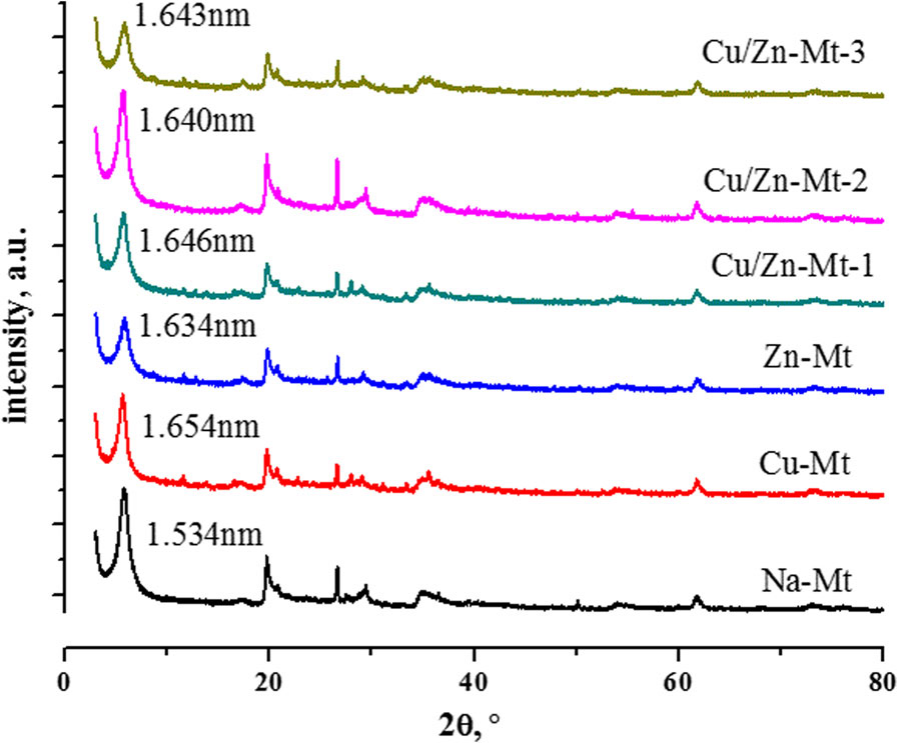
XRD patterns of modified Mt

XRD patterns were obtained to identify the intercalation of Cu^2+^ or and Zn^2+^ into Mt [20, 26]. The measured interlayer spacings d_001_ are shown in Fig. 1. Na-Mt displays a reflection at the 2θ value of 5.76°, which is assigned to d_001_ basal spacing of 1.534 nm. As for modified Mt, the reflection was emerged at lower 2θ values of 5.34°, 5.40°, 5.36°, 5.38°, 5.37° (Cu-Mt, Zn-Mt, Cu/Zn-Mt-1, Cu/Zn-Mt-2, Cu/Zn-Mt-3), corresponding to the increased d_001_ basal spacing of 1.654 nm, 1.638 nm, 1.649 nm, 1.641 nm, 1.645 nm. It can be presumed that these increases after Cu^2+^ or/and Zn^2+^ ion exchange are caused by a difference in the size of hydrated form between Cu^2+^/Zn^2+^ and Na^+^ ion, although ion radius of Cu and Zn (0.072 nm,0.074 nm, respectively) is smaller than that of sodium (0.095 nm). The sodium ions in Na-Mt have been exchanged with [Zn(H_2_O)_6_]^2+^ or/and [Cu(H_2_O)_6_]^2+^[27]. Moreover, no difference of d_001_ basal spacing was observed in modified Mt, which might be associated with similar ion radius between Cu^2+^ and Zn^2+^ .

**Fig. 2:**
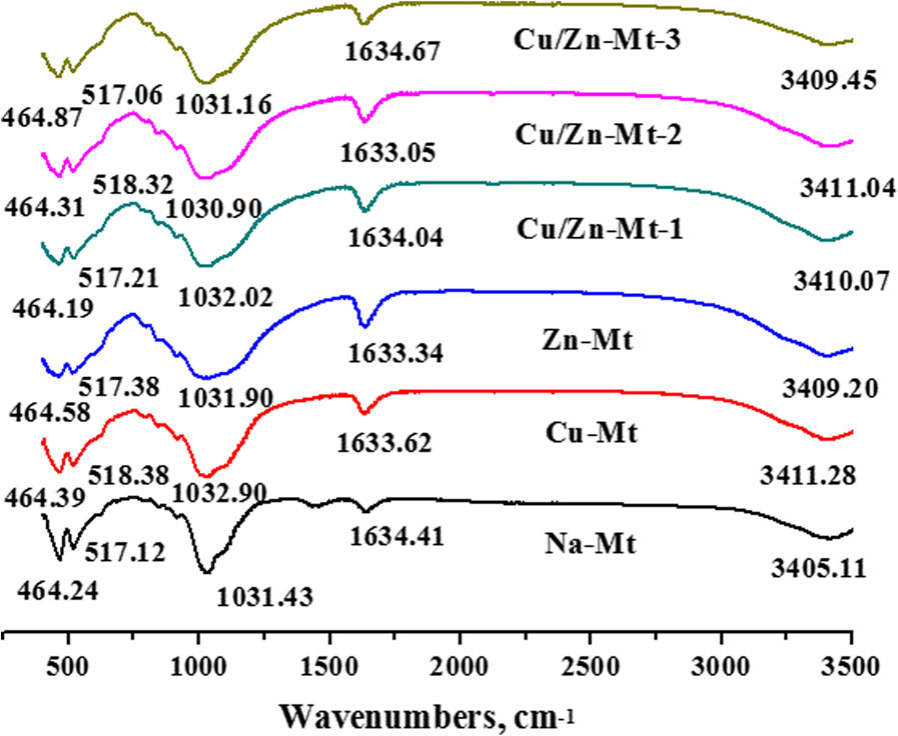
FT-IR spectra of modified Mt

The infrared spectra of the specimens were obtained by the KBr method using an FTIR spectrometer and demonstrated in Fig. 2. Characteristic bands for Na-Mt are present around 3,405 cm^−1^ and 1,633 cm^−1^ (O-H stretching), 1,035 cm^−1^ (Si-O stretching), 529 cm^−1^ and 464 cm^−1^ (Si-O bending vibration) [28, 29]. As for modified Mt (Cu-Mt, Zn-Mt, Cu/Zn-Mt), the O-H stretching vibration band increased slightly, which can be attributed to the hydrated Cu^2+^/Zn^2+^ ions [26]. In addition, the positions of the Si-O bending vibrations remained basically unchanged at 518 cm^−1^ (Si-O-Al) and 466 cm^−1^ (Si-O-Si) for the Cu/Zn-Mt, which indicated that the presence of Zn and Cu in the hexagonal cavities didn’t affects this vibration in the experiment.

**Fig. 3:**
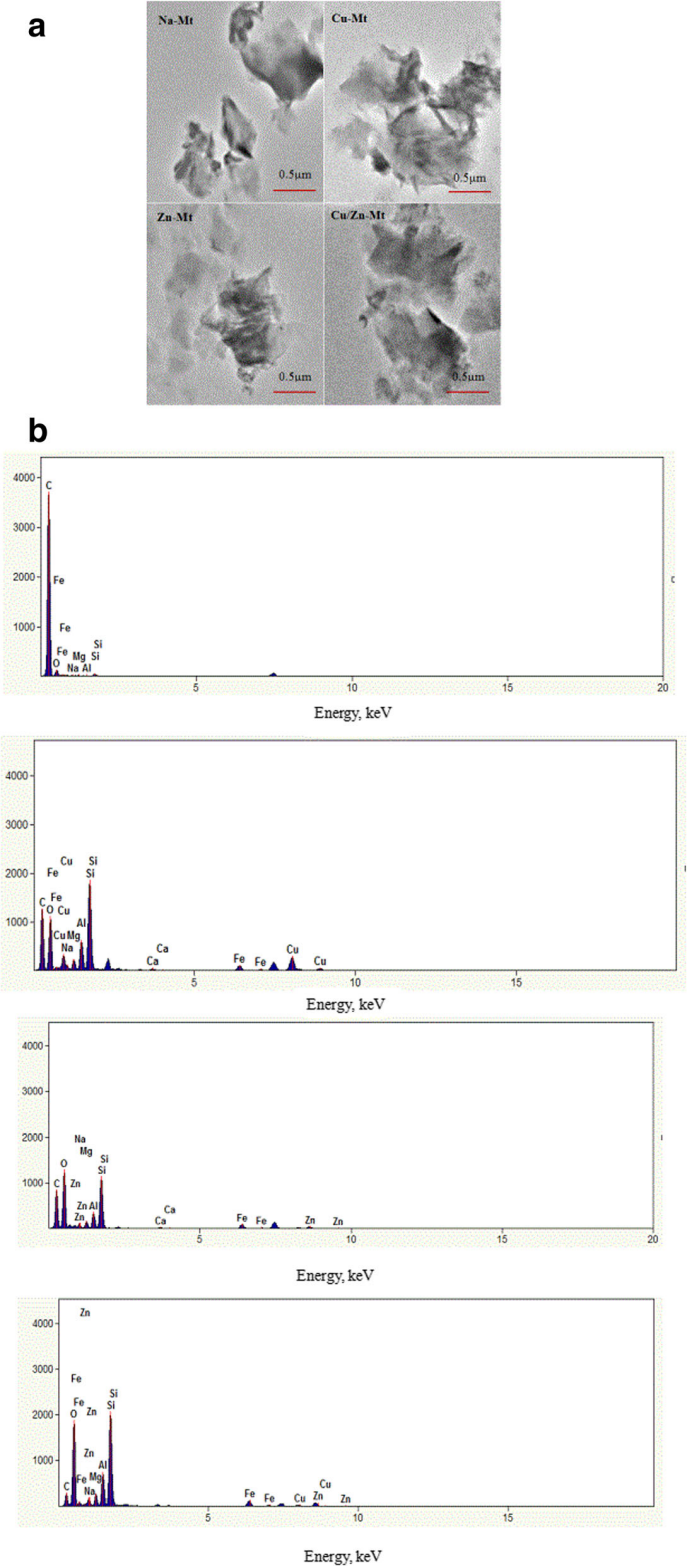
TEM and EDX spectrum of modified Mt

The TEM images reveal the internal structures of the Mt and modified Mt as presented in Fig. 3a. It was observed that the layered crystallites of Mt and modified Mt aggregated in large sized particles. Consistent with XRD, FTIR results, Cu or Zn loaded on the interlayers of Mt did not change the Mt’s layered structure. Moreover, Fig. 3b shows the EDX spectrum of the specimens, confirming the presence of Cu or Zn in modified Mt. Apart from elements of Na-Mt (Al, Si, O, C, Fe, Mg and Na), peak of Cu or/and Zn was visible in Cu–Mt, Zn–Mt and Cu/Zn-Mt. Peak of sodium is detected which indicates incomplete exchange with Cu or/and Zn.

### Antimicrobial assay of the specimens

The antimicrobial activity of the specimens was investigated in Table 1. In the case of Na-Mt, no antimicrobial activity was detected against the microorganisms (the MIC against the three kinds of microorganisms were all more than 10,000 mg/L). Similar results have also been reported that Mt with large specific surface area could adhere to bacteria by electrostatic forces, but showed no bacteriostatic activity [16, 30]. Moreover, results showed *E. coli* was more sensitive to modified Mt compared with *S. aureus* and *C. albicans.* One of the possible explanations of that difference in sensitivity is the different characteristics of the cell surfaces. It has been reported that the negative charge on the cell surface of gram-negative bacteria was higher than on gram-positive bacteria [12, 31]*.* Due to a higher negative charge on cell surface, the interaction between *E. coli* and Mt (positively charged in the interlayer) was definitely stronger than that of *S. aureus* and *C. albican*s, which could facilitate contact of Cu^2+^ and Zn^2+^ with bacterial cells wall and thus enable their damaging effect to the bacteria.

**Table 1:** The antimicrobial activity of modified Mt

Sample	MIC, mg/L		
	*E. coli*	*S. aureus*	*C. albicans*
Na-Mt	>10,000	>10,000	>10,000
Cu-Mt	411.63	657.78	1,315.56
Zn-Mt	823.26	1,315.56	2631.12
Cu/Zn-Mt-1	328.89	411.63	823.26
Cu/Zn-Mt-2	328.89	411.63	823.26
Cu/Zn-Mt-3	411.63	657.78	1,315.56

In addition, the antimicrobial effect was detected in Zn-Mt, Cu-Mt and Cu/Zn-Mt treatments. Compared with Zn-Mt, Cu-Mt displayed higher antibacterial and antifungal activity, which was corresponded to the previous results [10, 12]. The antibacterial and antifungal properties of Zn-Mt or Cu-Mt could been attributed to the attraction, by electrostatic forces, of the negatively charged membrane of the bacteria to the surface of the Mt, where the positive charged Cu or Zn ions kills the bacteria or renders them unable to replicate [20, 32]. In the case of Cu/Zn-Mt, the antibacterial and antifungal activity has been improved compared with Cu-Mt and Zn-Mt. Cu/Zn-Mt showed obvious synergistic antimicrobial effect. Similar to our findings, it was reported that compared with Chitosan/Ag and CS/ZnO, Chitosan/Ag/ZnO composite displayed excellent antimicrobial activities against *B. subtilis*, *E. coli*, *S. aureus*, *Penicillium*, *Aspergillus*, *Rhizopus* and *yeast* [33]. Moreover, it was found that Zn^2+^-Ce^3+^ loaded Mt presented much higher antibacterial and antifungal efficiency than Zn^2+^ loaded Mt and Ce^3+^ loaded Mt [17]. A recent review indicated that different metals caused discrete and distinct types of injuries to microbial cells as a result of oxidative stress, protein dysfunction or membrane damage [34]. It has been reported that toxicity associated with Cu might be due to impaired membrane function and reactive oxygen species (ROS) mediated cellular damage [35]. Zn could initiate bacteriostasis though oxidation of cellular thiols or damaging Fe–S-containing dehydratases in vitro independently of ROS and inhibit these enzymes activity [36]. Therefore, we speculated that different antimicrobial mechanism might result in more effective damage to bacteria, which might require further study. What is more, the antibacterial and antifungal properties of Cu/Zn-Mts varied with different Cu/Zn atomic ratios. Compared with Cu/Zn-Mt-3, Cu/Zn-Mt-1 and Cu/Zn-Mt-2 showed higher antibacterial and antifungal activity. So the study was especially focused on the structure and properties of Cu/Zn-Mt-2 in the following research.

### Particle size, surface properties and antimicrobial activity of the Cu/Zn-Mt-2

**Fig. 4:**
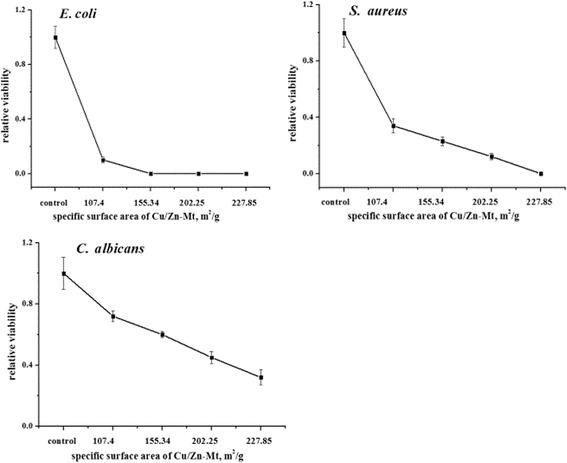
The antimicrobial activity of Cu/Zn-Mt-2 with different specific surface area

The effect of particle size on the antimicrobial activity of Cu/Zn-Mt (Cu/Zn-Mt-2) was seen in Fig. 4. The antimicrobial activity increased with the enhancement of the specific surface area when the particle size decreased, which suggested that the overall antimicrobial effect is not only related to the presence and quantity of metal ions but also is affected by the surface characteristics of the modified Mt [16]. It was reasonable to state that the binding of Cu/Zn-Mt particles to the bacteria depended on the surface area available for interaction. Smaller particles having the larger surface area available for interaction would give higher antimicrobial effect than the larger particles [35].

### Cytotoxicity assay of the Cu/Zn-Mt-2

**Fig. 5:**
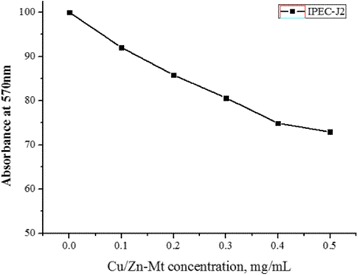
The cytotoxicity of Cu/Zn-Mt-2

In order to utilize the antimicrobial effect of Cu/Zn-Mt for therapeutic purposes, it is absolutely essential to perform a cytotoxicity study (Fig. 5). Cu/Zn-Mt-2 (0.1 mg/mL) exhibited a slight cytotoxicity (~10%) to IPEC-J2 cell within 24 h incubation by MTT assays. Cu/Zn-Mt-2 of higher concentration (0.3 mg/mL) led to increased cytotoxicity (~20%) within 24 h. Overall, the concentrations of the cell population showed stabilization at concentrations beyond the MIC value. This may be explained as follows, lower concentration of Cu or Zn had minimal adverse effect on cells in vitro [32, 37]. Moreover, Mt has been regarded as a dermatological and gastrointestinal protector by attaching to the cells and absorbing any toxic molecules due to their high absorptive capacity, which played an important role in reducing toxicity of Cu/Zn-Mt-2 [38]. Therefore, Cu/Zn-Mt was good inorganic antimicrobial materials with slight cytotoxicity.

## Conclusion

Cu/Zn-Mt with different Cu/Zn ratios could be prepared by an ion-exchange reaction and showed synergistic antimicrobial effect and relatively low cell toxicity. Moreover, the antimicrobial activity of Cu/Zn-Mt was correlated with its specific surface area and Cu/Zn ratios. Therefore, Cu/Zn-Mt holds great promise for applications in animal husbandry.

## Abbreviations

Cu/Zn-Mt: Copper/zinc loaded montmorillonite
Cu-Mt: Copper loaded montmorillonite
Mt: Montmorillonite
Zn-Mt: Zinc loaded montmorillonite
